# Spectrum of congenital heart disease in Nepal from 2002–2022: A systematic review and meta‐analysis

**DOI:** 10.1002/hsr2.1147

**Published:** 2023-03-13

**Authors:** Oshan Shrestha, Niranjan Thapa, Sagun Karki, Prechha Khanal, Prashant Pant, Arun Neopane

**Affiliations:** ^1^ Nepalese Army Institute of Health Sciences, College of Medicine Kathmandu Nepal; ^2^ Gandaki Medical College Teaching Hospital and Research Centre Pokhara Kaski Nepal; ^3^ Department of Medicine Star Hospital Kathmandu Nepal; ^4^ Department of Paediatrics Nepalese Army Institute of Health Sciences Kathmandu Nepal

**Keywords:** atrial septal defect, congenital heart disease, ventricular septal defect

## Abstract

**Background:**

Congenital heart diseases are recognized as public health concerns worldwide and Nepal is no exception. This study aims to study congenital heart disease in Nepal on grounds of burden, commonest type, common presentations, and associated noncardiac anomalies so that its spectrum can be known for prompt diagnosis and adoption of screening protocols.

**Methods:**

Relevant articles were searched in electronic databases using appropriate search terms and Boolean operators. Data were extracted in Excel and analyzed in Comprehensive Meta‐Analysis Software. The proportion was used as an effect measure and a fixed or random‐effect model was used as per the heterogeneity. Forest plots were used to give visual feedback.

**Results:**

A total of nine studies were included in the qualitative and quantitative synthesis after a rigorous screening of imported studies. The prevalence of congenital heart disease was 0.7% (Proportion: 0.007; CI: 0.001–0.035; I^2^: 99.263%). The burden of atrial septal defect was 32.1%, ventricular septal defect was 31.1%, patent ductus arteriosus was 12.6% and Tetralogy of Fallot was 7.3%. The most common presentations were respiratory tract infection (54.7%), developmental delay (49.8%), difficulty in breathing (44.5%), failure to thrive (17.1%), and cyanosis (15.9%).

**Conclusions:**

The prevalence of congenital heart disease in Nepal was 0.7% and the frequency of male patients was higher. The atrial septal defect was the commonest on the whole, while, Tetralogy of Fallot was the commonest among cyanotic variety. Respiratory tract infection was frequently seen at presentation and the most commonly associated noncardiac anomaly was the cleft palate.

## 1 BACKGROUND

Structural abnormalities of the heart or its great vessels, occurring during fetal development, which are of functional significance, are studied and grouped as congenital heart disease (CHD).^1^, ^2^ These abnormalities affect about 0.8%–1.2% of children per year and is one of the leading cause of neonatal mortality.^3^ CHD can be grouped into two types based on the effect of the defect on the oxygen saturation of the body, namely, cyanotic CHD and noncyanotic CHD.^1^, ^4^ Common noncyanotic or acyanotic CHD include atrial septal defect (ASD), ventricular septal defect (VSD), patent ductus arteriosus (PDA) while common cyanotic CHD include total anomalous pulmonary venous connection (TAPVC), transposition of great arteries (TGA), tetralogy of Fallot (ToF). Among these, the ones that require intervention in the first year of life only are grouped as critical congenital heart disease (CCHD).^5^ Among CHD, about 25% are CCHD and these have high morbidity and mortality.^1^, ^5^ CHD is recognized as one of the major public health concerns worldwide and Nepal is no exception. Few studies have been conducted in Nepal on CHD but there is no concrete data. This study aims to systematically review and do a meta‐analysis on the topic. The burden of CHD will be the primary focus of this study while, the commonest type of CHD in Nepal, common presenting complaints, and associated noncardiac anomalies will also be studied. This will establish concrete data and its spectrum can be known for prompt diagnosis and adoption of screening protocols in newborns.

## 2 METHODS

This study is in line with Meta‐analysis of Observational Studies in Epidemiology (MOOSE) guidelines.^6^

### 2.1 Protocol registration

The protocol followed in this systematic review and meta‐analysis registered in the International prospective register of systematic reviews (PROSPERO) with PROSPERO ID CRD42022366747.^7^

### 2.2 Search strategy

We explored electronic databases like (PubMed, PubMed Central, Scopus, Embase and Nepal Journals Online) for relevant articles by using appropriate search terms and Boolean operators as (“congenital heart disease” OR CHD OR “cyanotic heart disease” OR “acyanotic heart disease”) AND (prevalence OR pattern OR spectrum) AND (Nepal). A time filter was used to search articles from the year 2002 to October, 2022. Details of the search and the results obtained from each database is available in Supporting Information: File 1.

### 2.3 Inclusion criteria and exclusion criteria

This study intended to include all published articles (except for editorial, review articles, viewpoints, and commentaries) that have reported data on CHD of patients below 18 years of age, in Nepal.

### 2.4 Study selection

All the relevant studies were imported to Covidence^8^ and screening was done by two independent authors. Any conflicts that rose were resolved by the third reviewer. The role of the primary screener and conflict resolver were exchanged in the title‐and‐abstract screening phase and full‐text screening phase.

### 2.5 Data curation

A data collection tool was prepared in Excel and the same tool was used to extract data from included nine studies. The data extracted in Excel was peer‐reviewed for correction of mistakes and refinement of the data. The data collection tool contained headings like author/s, study year, study design, study center, study address, sample size, study Population, total CHD patients, type of cardiac defect, presenting complaints, and associated noncardiac anomaly. While taking the data of the type of cardiac lesion from studies, the frequency of cardiac defects given in combination with noncardiac anomaly (like ASD + hernia) was treated as isolated cardiac lesions, whereas cardiac defects given in combination with other cardiac anomalies (like ASD + VSD) were given respective separate heading during data curation.

### 2.6 Data synthesis

Statistical analysis was performed by using Comprehensive Meta‐Analysis Software (CMA) version 3.^9^ Proportion was used as the effect measure, I^2^ test was used for heterogeneity, and fixed or random‐effect model was used as per the heterogeneity.^10^ Forest plot was used to give visual feedback.

### 2.7 Risk of bias assessment

Risk of bias assessment of the individual study was performed using the Joanna Briggs Institute (JBI) critical appraisal tool.^11^ Assessment of bias is shown in Table 1.

**Table 1:** JBI critical appraisal for bias assessment.

JBI Questionnaire	Chapagain and colleagues	Singh and colleagues	Joshi and colleagues	Man Bahadur and colleagues	Mishra and colleagues	Ramachandaran and colleagues	Shah and colleagues	Shah and colleagues	Shrestha and colleagues
Was the sample frame appropriate to address the target population?	Yes	Yes	Yes	Yes	Yes	Yes	Yes	Yes	Yes
Were study participants sampled in an appropriate way?	N/A	N/A	N/A	Yes	N/A	N/A	N/A	N/A	Yes
Was the sample size adequate?	Yes	No	Yes	Yes	Yes	Yes	Yes	Yes	Yes
Were the study subjects and the setting described in detail?	Yes	Yes	Yes	Yes	Yes	Yes	Yes	Yes	Yes
Was the data analysis conducted with sufficient coverage of the identified sample?	Yes	Yes	Yes	Yes	Yes	Unclear	Unclear	Unclear	Unclear
Were valid methods used for the identification of the condition?	Yes	No/unclear	No/unclear	No/unclear	No/unclear	No/unclear	No/unclear	Yes	Yes
Was the condition measured in a standard, reliable way for all participants?	Unclear	Yes	Unclear	Unclear	Unclear	Unclear	Unclear	Yes	Unclear
Was there appropriate statistical analysis?	Yes	Yes	Yes	Yes	Yes	Yes	Yes	Yes	Yes
Was the response rate adequate, and if not, was the low response rate managed appropriately?	Unclear	Unclear	Unclear	Yes	Unclear	Unclear	Unclear	Unclear	Unclear
Overall appraisal	Include	Include	Include	Include	Include	Include	Include	Include	Include

### 2.8 Subgroup analysis

Subgroup analysis was done for sex distribution and among different subtypes of CHD.

### 2.9 Sensitivity analysis

Sensitivity analysis was performed by re‐running analysis by excluding individual studies to check the impact of the study on the overall result.

## 3 RESULTS

**Figure 1:**
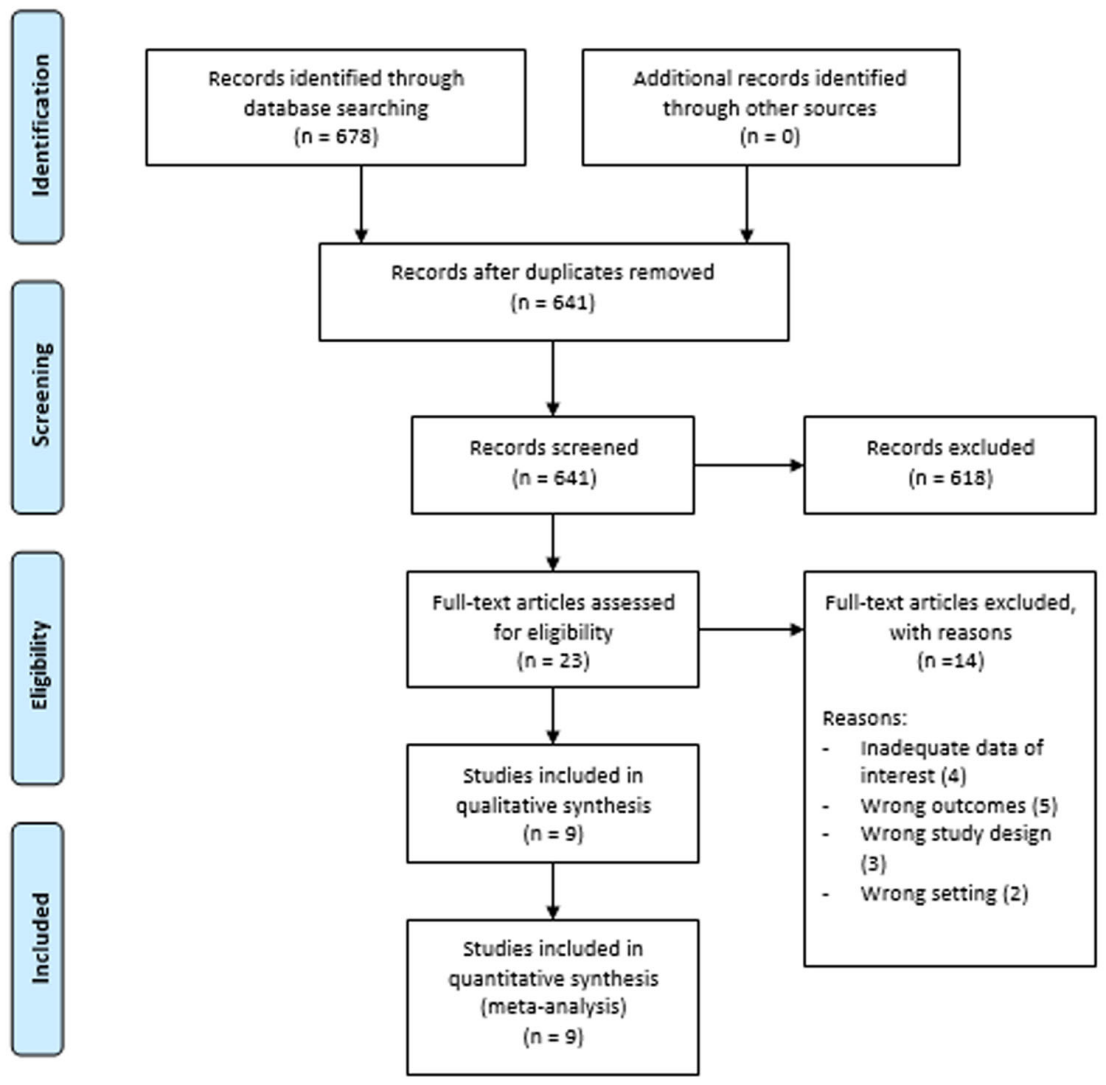
PRISMA flow diagram.

Search results of electronic databases yielded 678 studies, from which 37 duplicates were removed. Title and abstract screening were done for 641 studies out of which 618 studies were excluded. The full‐text screening was done for 23 studies and in this phase, 14 studies were excluded with reasons. A total of nine studies were included in the qualitative and quantitative synthesis. Details are shown in Figure 1.

### 3.1 Qualitative synthesis

Summary of the details of the included studies are shown in Table 2.

**Table 2:** Summary of included studies.

Study	Study design	Sample size	Total CHD	Study setting	Outcome
Chapagain et al.^12^ (2016)	Prospective, cross‐sectional study	831	85	Kanti Children's Hospital	Presenting complaints of patients with CHD, type of structural defect among patients of CHD, associated noncardiac anomaly, maternal history and mode of delivery
Singh et al.^13^ (2015–2017)	Prospective, cross‐sectional study	9473	35	Dhulikhel Hospital	Prevalence of congenital anomaly, frequency of different system involved and types of congenital anomaly, comparison between study group and reference group on sociodemographic and risk factors
Joshi et al.^14^ (2014–2016)	Prospective, cross‐sectional study	144	144	Dhulikhel Hospital	Pattern of cardiac disease presenting at different age groups, frequency and types of CHD, RHD, pericardial disease, dilated cardiomyopathy
Man Bahadur et al.^15^ (2002)	Prospective, cross‐sectional study	9420	12	Community school	Prevalence of CHD and RHD, types of CHD and sex distribution of the patients
Mishra et al.^16^ (2017)	Prospective, cross‐sectional study	150	150	Shahid Gangalal National Heart Centre	Age distribution, types of defect among CHD, duration of illness, assessment of health‐related quality of life
Ramachandaran et al.^17^ (2000–2002)	Prospective, cross‐sectional study	55	55	Manipal Teaching Hospital	Types of cardiac disease and age distribution, nature of cardiac defects, age of onset of symptoms, presenting symptoms
Shah et al.^18^ (2006)	Retrospective, cross‐sectional study	14,461	84	B.P Koirala Institute of Health Sciences	Age and sex distribution for CHD, presenting symptoms, frequency of type of cardiac defects
Shah et al.^19^ (2015–2016)	Prospective, cross‐sectional study	254	254	B.P Koirala Institute of Health Sciences	Sex distribution among types of CHD, frequency of type of lesion
Shrestha et al.^20^ (2017–2018)	Prospective, cross‐sectional study	2456	12	Kathmandu Medical College	Prevalence of congenital malformation, distribution of congenital malformation in different systems, frequency of various types of congenital defects

Abbreviation: CHD, congenital heart disease.

### 3.2 Quantitative synthesis

#### 3.2.1 Prevalence of CHD

**Figure 2:**
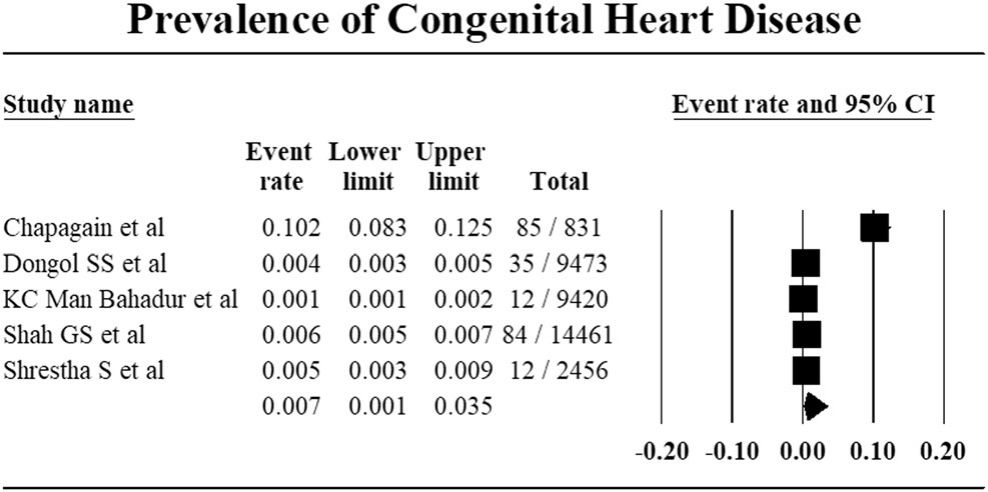
Forest plot showing the prevalence of congenital heart disease.

Pooling data using a random effect model from five studies reporting the total sample population and the total number of CHD cases, the prevalence was found to be 0.7% (Proportion: 0.007; CI: 0.001–0.035; I^2^: 99.263%) (Figure 2). Sensitivity analysis of the prevalence of CHD was carried out by excluding individual studies, which showed no significant differences in the result, details given in Supporting Information: File 2.

#### 3.2.2 Sex distribution

Among the included studies, three studies mentioned the data of sex distribution and pooling of that data showed that among the CHD cases 52.5% (Proportion: 0.525; CI: 0.409–0.639; I^2^: 62.472%) were males and 47.5% (Proportion: 0.475; CI: 0.361–0.591; I^2^: 62.472%) were females. Forest plot is given in Supporting Information: File 2.

#### 3.2.3 Atrial septal defect among CHD

**Figure 3:**
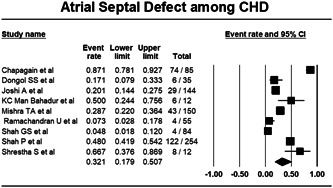
Forest plot showing burden of atrial septal defect among congenital heart disease.

Pooling data from nine studies, that reported the data of ASD using random effect model, showed that among the cases of CHD in Nepal, 32.1% (Proportion, 0.321%; CI, 0.179–0.507; I^2^, 94.256%) are ASD cases (Figure 3).

#### 3.2.4 Ventricular septal defect among CHD

**Figure 4:**
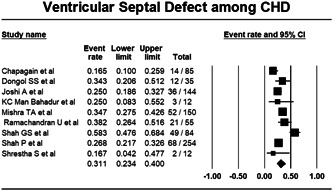
Forest plot showing burden of ventricular septal defect among congenital heart disease.

Among the nine studies reporting the data of VSD, pooling of data using random effect model showed that 31.1% (Proportion: 0.311; CI: 0.234–0.400; I^2^: 81.152%) among CHD cases are of VSD (Figure 4).

#### 3.2.5 Patent ductus arteriosus among CHD

**Figure 5:**
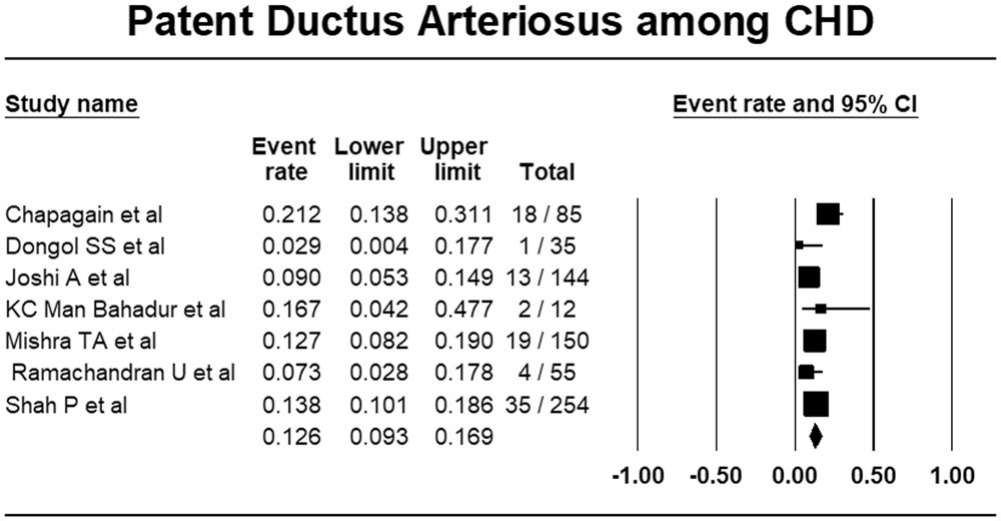
Forest plot showing patent ductus arteriosus among congenital heart disease.

Among studies reporting PDA cases, pooling of the data from seven studies data using fixed effect model showed the burden of PDA to be 12.6% (Proportion: 0.126; CI: 0.093–0.169; I^2^: 46.593%) (Figure 5).

#### 3.2.6 Tetralogy of Fallot among CHD

**Figure 6:**
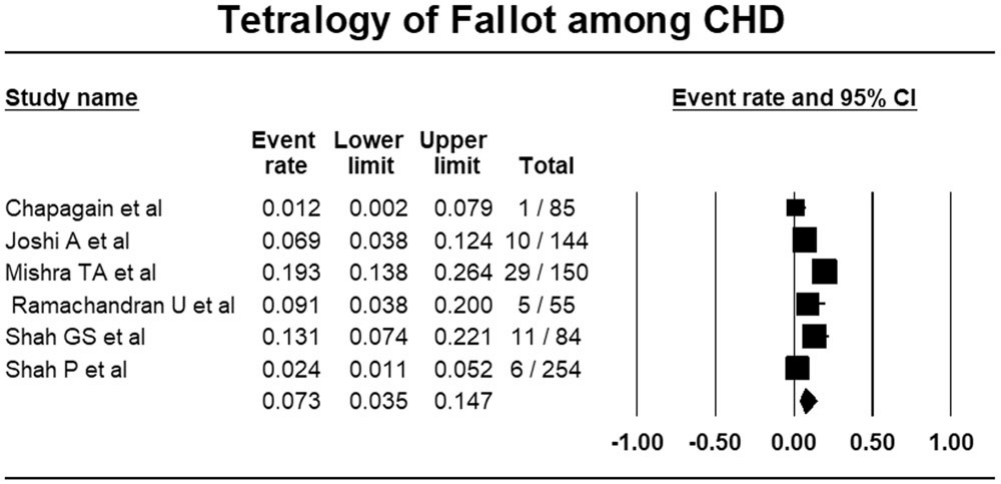
Forest plot showing tetralogy of Fallot among congenital heart disease.

Pooling data from six studies reporting data of Tetralogy of Fallot (ToF) using random effect model showed that 7.3% (Proportion: 0.073; CI: 0.035–0.147; I^2^: 85.381%) of cases were of ToF (Figure 6).

#### 3.2.7 Pulmonary stenosis among CHD

Pooling data from five studies using fixed effect model showed that 3.9% (Proportion: 0.039; CI: 0.025–0.060; I^2^: 0.000%) cases were of pulmonary stenosis. Forest plot is given in Supporting Information: File 2.

#### 3.2.8 Aortic stenosis among CHD

Pooling data from three studies using fixed effect model showed that 3.1% (Proportion: 0.031; CI: 0.015–0.064; I^2^: 0.000%) cases were of aortic stenosis. Forest plot is given in Supporting Information: File 2.

#### 3.2.9 Dextrocardia among CHD

Pooling data from three studies using fixed effect model showed that 2.3% (Proportion: 0.023; CI: 0.010–0.051; I^2^: 0.000%) cases were of dextrocardia. Forest plot is given in Supporting Information: File 2.

#### 3.2.10 Coarctation of aorta among CHD

Data of coarctation of aorta was available in two studies and pooling of data using fixed effect model showed that 1.5% (Proportion: 0.015; CI: 0.005–0.046; I^2^: 0.000%) cases were of coarctation of aorta. Forest plot is given in Supporting Information: File 2.

#### 3.2.11 Total anomalous pulmonary venous connection among CHD

Pooling of the studies reporting data of TAPVC using fixed effect model showed that 2.0% (Proportion: 0.020; CI: 0.012–0.036; I^2^: 0.000%) of the cases were of TAPVC. Forest plot is given in Supporting Information: File 2.

#### 3.2.12 Double outlet right ventricle among CHD

Three studies reported the data of DORV and pooling of the data using fixed effect model showed its burden to be 3.6% (Proportion: 0.036; CI: 0.022–0.058; I^2^: 11.341%). Forest plot is given in Supporting Information: File 2.

#### 3.2.13 Transposition of the great arteries among CHD

Data of TGA was available in six studies and pooling of data using fixed effect model showed its burden to be 2.4% (Proportion: 0.024; CI: 0.014–0.040; I^2^: 0.000%). Forest plot is given in Supporting Information: File 2.

#### 3.2.14 Truncus arteriosus among CHD

Two studies reported the data of truncus arteriosus and pooling of data using fixed effect model showed its burden to be 0.9% (Proportion: 0.009; CI: 0.003–0.028; I^2^: 6.423%). Forest plot is given in Supporting Information: File 2.

#### 3.2.15 Complex cardiac defect among CHD

Five studies reported complex cardiac defect and pooling of data using random effect model showed its burden to be 12.5% (Proportion: 0.125; CI: 0.062–0.236; I^2^: 76.070%). Forest plot is given in Supporting Information: File 2.

#### 3.2.16 Common presenting complaints

The most common presenting symptoms were found to be respiratory tract infection, developmental delay, difficult breathing, failure to thrive, and cyanosis in decreasing order. Details are mentioned in Table 3 and forest plots are given in Supporting Information: File 2.

**Table 3:** Common presenting problems of the patients of CHD.

Symptoms	Percentage	Proportion (confidence interval)	Heterogeneity (I^2^)	Effect model
Respiratory infection	54.7%	0.547 (CI: 0.463−0.627)	0.000%	Fixed
Developmental delay	49.8%	0.498 (CI: 0.023−0.977)	98.159%	Random
Difficult breathing	44.5%	0.445 (CI: 0.197−0.724)	93.634%	Random
Failure to thrive	17.1%	0.171 (CI: 0.084−0.317)	68.807%	Random
Cyanosis	15.9%	0.159 (CI: 0.092−0.262)	54.810%	Random

Abbreviation: CHD, congenital heart disease.

#### 3.2.17 Associated noncardiac anomaly

**Figure 7:**
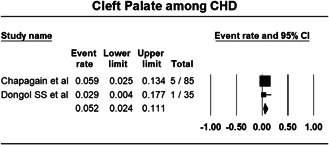
Forest plot showing cleft palate among cases of congenital heart disease.

Various noncardiac anomaly being associated with CHD were given in included studies, but meta‐analysis could be performed only for cleft palate. Cleft palate was found among 5.2% (Proportion: 0.052; CI: 0.024‐ 0.111; I^2^: 0.000%) cases of CHD (Figure 7).

## 4 DISCUSSION

Congenital heart disease poses a great risk of morbidity/mortality and the entity is frequently encountered in the community. The present study, with pediatric age‐group as study population, quantified prevalence of congenital heart disease in Nepal to be 0.7% (7 per 1000 individuals), by using data from nine different studies done across various regions of the country. Need for this study became crucial as there was no meta‐analysis data that could be representative for the nation. Nepal is one of the lower‐middle‐income countries (LMIC) and can offer only the bare minimum to the citizens in the health sector. In such conditions, screening and reporting of the cases are not optimal. The burden of CHD that has been quantified in various studies is not the ground reality, and this can be said as the institutional delivery rate in Nepal is just 58% and the burden of CHD in the neighboring countries (India‐1.01% and China‐1.65%) and the whole Asia (0.93%) is more.^21^, ^22^, ^23^, ^24^ This speculation can only be accepted or discarded after thorough and extensive further study.

Among the categories of congenital structural abnormality of heart, acyanotic or noncyanotic CHD are the most common ones worldwide^24^ and the findings of our study is in line with it. Ventricular septal defect is encountered as the most common one, however, this study found that atrial septal defect was commonest in Nepal. Talking about the commonest subtypes of CHD found worldwide are ventricular septal defect, atrial septal defect, patent ductus arteriosus, pulmonary stenosis, Tetralogy of Fallot, coarctation of aorta, transposition of great arteries, and aortic stenosis. In our study also, these subtypes were constant as the commonest subtypes. Complex cardiac defects also had a higher frequency among subtypes of CHD. Discussing another entity, subtypes like tetralogy of Fallot, total anomalous pulmonary venous connection, hypoplastic left heart syndrome, transposition of great arteries, tricuspid atresia, pulmonary atresia, and truncus arteriosus are collectively called critical congenital heart disease.^25^ Critical congenital heart disease are names such as they need prompt intervention in the first year of life and have the highest mortality rate.^1^ In this study, it is found that 12.6% of CHD cases are critical congenital heart diseases. However, it is estimated that about 25% of the total CHD cases are critical CHD.^1^ This also raises the suspicion of underreporting due to the geographical and economical state of the country. Since the mortality of the condition is high, every neonate should be screened for the condition. Among the different means of screening, pulse oximetry is also effective one^1^, ^25^ that suits Nepal, where the question of availability and affordability of imaging modalities are prevalent.

Presentation of the patient may vary in congenital heart disease due to the variability in structural defect and can be symptoms of the condition itself or complications of the condition. In this study, the most common presenting complaint of CHD was found to be respiratory infection followed by developmental delay, difficulty in breathing, failure to thrive, and cyanosis. The age of onset of symptoms could not be studied due to the unavailability of the data but medical literature suggests that the symptoms normally do not develop until early childhood or teenage years.^26^ Congenital cardiac defect is a structural abnormality and it may be present in an individual solely or can come with other noncardiac abnormalities. Pooling the data from included studies showed that the most commonly associated noncardiac anomaly is cleft‐palate. This finding is consistent with the findings of other studies.^27^, ^28^

Nepal needs to adopt cost‐effective screening methods or protocols to screen neonates for CHD to avoid delays in diagnosis and intervention. This study recommends the diagnosis of structural heart defects before the development of clinical signs early in life through screening and government‐level intervention. At the present, there is no established culture of screening for structural heart disease early in life, but we can adopt pulse oximetry screening for early detection of CCHD until concerned authorities, government or concerned medical society formulates a protocol that is best suited for Nepal.

This study had its limitations, this study took patients of age below 18 years to pool data and included studies were mostly from main city areas that did not represent data from all corners of the country. The heterogeneity among studies was due to differences in sample size and inclusion age criteria. Also, there is a lack of general population level screening of CHD in early life which risks cases being underreported and the calculated prevalence being underestimation.

## 5 CONCLUSION

The prevalence of congenital heart disease in Nepal was 7 per 1000 individuals in pediatric age group and the frequency of males was higher compared to females. Noncyanotic heart disease was more common, the atrial septal defect being the commonest of all, while Tetralogy of Fallot was commonest among cyanotic heart disease. CHD should also be in the minds of healthcare providers when patients present with respiratory infection and cleft palate as these are more common than cyanosis. Also, cost‐effective screening protocols are warranted to avoid delays in diagnosis and intervention.

## AUTHOR CONTRIBUTIONS

Oshan Shrestha, Niranjan Thapa, and Sagun karki were involved in the concept and design of the study. Oshan Shrestha, Niranjan Thapa, Sagun karki, Prechha Khanal, and Prashant Pant were involved in the literature search, screening, and data extraction. Oshan Shrestha and Arun Neopane were involved in data analysis and interpretation. Oshan Shrestha, Niranjan Thapa, and Sagun karki drafted the initial manuscript and all authors were involved in revising and approving the final version. Arun Neopane guided throughout the study.

## CONFLICT OF INTEREST STATEMENT

The authors declare no conflict of interest.

## TRANSPARENCY STATEMENT

The lead author Oshan Shrestha affirms that this manuscript is an honest, accurate, and transparent account of the study being reported; that no important aspects of the study have been omitted; and that any discrepancies from the study as planned (and, if relevant, registered) have been explained.

## Supporting information


**Supplementary information**.

**Supplementary information**.

## Data Availability

All the data curated in this study is available from the corresponding author on reasonable request.
