# Supplementary material for: Spectrum of congenital heart disease in Nepal from 2002–2022: A systematic review and meta‐analysis
Source: Health Sci Rep. 2023 Mar 13;6(3):e1147. doi: 10.1002/hsr2.1147 (PMC10011045; doi:10.1002/hsr2.1147)
Supplement: Supplementary file 1 — Supplementary information. [file HSR2-6-e1147-s002.docx]

Search details:

EMBASE:

('congenital heart disease'/exp OR 'congenital heart disease' OR chd OR 'cyanotic heart disease'/exp OR 'cyanotic heart disease' OR 'acyanotic heart disease'/exp OR 'acyanotic heart disease') AND ('prevalence'/exp OR prevalence OR pattern OR 'spectrum'/exp OR spectrum) AND ('nepal'/exp OR nepal) AND [2002-2022]/py

Time filter: 2002-2022

Hits: 50

Link: <https://www.embase.com/#advancedSearch/resultspage/history.1/page.1/25.items/orderby.date/source>.

SCOPUS:

("congenital heart disease" OR CHD OR "cyanotic heart disease" OR "acyanotic heart disease") AND (prevalence OR pattern OR spectrum) AND (Nepal)

Time filter: 2002-2022

Hits: 14

Link: <https://www.scopus.com/results/results.uri?sort=plf-f&src=s&st1=%28%22congenital+heart+disease%22+OR+CHD+OR+%22cyanotic+heart+disease%22+OR+%22acyanotic+heart+disease%22%29+AND+%28prevalence+OR+pattern+OR+spectrum%29+AND+%28Nepal%29&sid=4e7a8f2c62f8c19f2144183b7fa88587&sot=b&sdt=b&sl=159&s=TITLE-ABS-KEY%28%28%22congenital+heart+disease%22+OR+CHD+OR+%22cyanotic+heart+disease%22+OR+%22acyanotic+heart+disease%22%29+AND+%28prevalence+OR+pattern+OR+spectrum%29+AND+%28Nepal%29%29&origin=searchbasic&editSaveSearch=&yearFrom=2002&yearTo=2022>

PUBMED:

("congenital heart disease" OR CHD OR "cyanotic heart disease" OR "acyanotic heart disease") AND (prevalence OR pattern OR spectrum) AND (Nepal)

Time filter: 2002-2022

Hits: 20

Link: <https://pubmed.ncbi.nlm.nih.gov/?term=%28%22congenital+heart+disease%22+OR+CHD+OR+%22cyanotic+heart+disease%22+OR+%22acyanotic+heart+disease%22%29+AND+%28prevalence+OR+pattern+OR+spectrum%29+AND+%28Nepal%29&filter=years.2002-2022>

PMC

("congenital heart disease"[All Fields] OR "chd"[All Fields] OR "cyanotic heart disease"[All Fields] OR "acyanotic heart disease"[All Fields]) AND (("epidemiology"[Subheading] OR "epidemiology"[All Fields] OR "prevalence"[All Fields] OR "prevalence"[MeSH Terms]) OR pattern[All Fields] OR "spectrum"[All Fields]) AND ("nepal"[MeSH Terms] OR "nepal"[All Fields]) AND ("2002/01/01"[PubDate] : "2022/12/31"[PubDate])

Time filter: 2002-2022

Hits: 586

Link: <https://www.ncbi.nlm.nih.gov/pmc?term=%28%22congenital%20heart%20disease%22%5BAll%20Fields%5D%20OR%20%22chd%22%5BAll%20Fields%5D%20OR%20%22cyanotic%20heart%20disease%22%5BAll%20Fields%5D%20OR%20%22acyanotic%20heart%20disease%22%5BAll%20Fields%5D%29%20AND%20%28%28%22epidemiology%22%5BSubheading%5D%20OR%20%22epidemiology%22%5BAll%20Fields%5D%20OR%20%22prevalence%22%5BAll%20Fields%5D%20OR%20%22prevalence%22%5BMeSH%20Terms%5D%29%20OR%20pattern%5BAll%20Fields%5D%20OR%20%22spectrum%22%5BAll%20Fields%5D%29%20AND%20%28%22nepal%22%5BMeSH%20Terms%5D%20OR%20%22nepal%22%5BAll%20Fields%5D%29%20AND%20%28%222002/01/01%22%5BPubDate%5D%20%3A%20%222022/12/31%22%5BPubDate%5D%29&cmd=DetailsSearch>

NepJol

(congenital heart disease" OR "cyanotic heart disease" OR "congenital cardiac defect" OR "acyanotic heart disease") AND (prevalence OR spectrum)

Time filter: 2002-2022 oct

Hits: 8

Link: <https://www.nepjol.info/index.php/index/search/index?query=%28congenital+heart+disease%22+OR+%22cyanotic+heart+disease%22+OR+%22congenital+cardiac+defect%22+OR+%22acyanotic+heart+disease%22%29+AND+%28prevalence+OR+spectrum%29&dateFromYear=2002&dateFromMonth=1&dateFromDay=1&dateToYear=2022&dateToMonth=10&dateToDay=31&authors=>
