# Supplementary material for: Spectrum of congenital heart disease in Nepal from 2002–2022: A systematic review and meta‐analysis
Source: Health Sci Rep. 2023 Mar 13;6(3):e1147. doi: 10.1002/hsr2.1147 (PMC10011045; doi:10.1002/hsr2.1147)

Prevalence of Congenital Heart Disease

Study name	Point	Lower limit	Upper limit
Chapagain et al	0.004	0.002	0.006
Dongol SS et al	0.008	0.001	0.057
KC Man Bahadur et al	0.010	0.002	0.061
Shah GS et al	0.007	0.001	0.067
Shrestha S et al	0.008	0.001	0.049
	0.007	0.001	0.035

Event rate (95% CI) with study removed

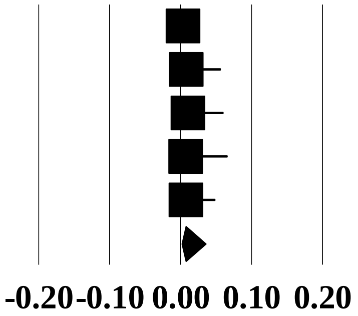

Proportion of males among CHD

Study name	Event rate	Lower limit	Upper limit	Total
KC Man Bahadur et al	0.250	0.083	0.552	3 / 12
Shah GS et al	0.607	0.499	0.705	51 / 84
Shah P et al	0.524	0.462	0.584	133 / 254
	0.525	0.409	0.639	

Event rate and 95% CI

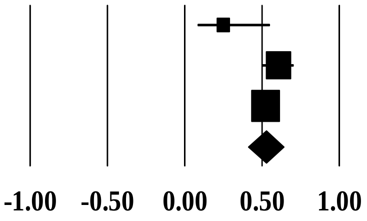

Proportion of females among CHD

Study name	Event rate	Lower limit	Upper limit	Total
KC Man Bahadur et al	0.750	0.448	0.917	9 / 12
Shah GS et al	0.393	0.295	0.501	33 / 84
Shah P et al	0.476	0.416	0.538	121 / 254
	0.475	0.361	0.591	

Event rate and 95% CI

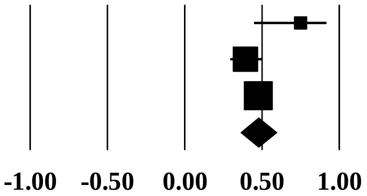

Pulmonary Stenosis among CHD

Study name	Event rate	Lower limit	Upper limit	Total
Chapagain et al	0.059	0.025	0.134	5 / 85
KC Man Bahadur et al	0.083	0.012	0.413	1 / 12
Mishra TA et al	0.020	0.006	0.060	3 / 150
Ramachandran U et al	0.055	0.018	0.156	3 / 55
Shah P et al	0.031	0.016	0.062	8 / 254
	0.039	0.025	0.060	

Event rate and 95% CI

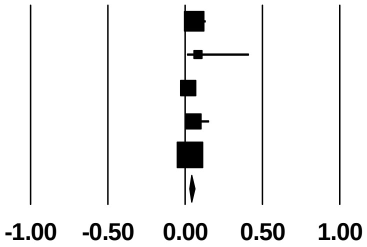

Aortic Stenosis among CHD

Study name	Event rate	Lower limit	Upper limit	Total
Dongol SS et al	0.057	0.014	0.202	2 / 35
Mishra TA et al	0.027	0.010	0.069	4 / 150
Ramachandran U et al	0.018	0.003	0.118	1 / 55
	0.031	0.015	0.064	

Event rate and 95% CI

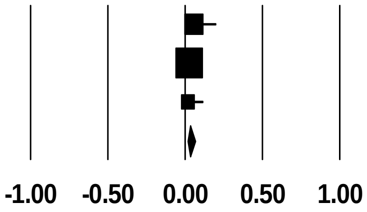

Coarctation of aorta among CHD

Study name	Event rate	Lower limit	Upper limit	Total
Joshi A et al	0.014	0.003	0.054	2 / 144
Ramachandran U et al	0.018	0.003	0.118	1 / 55
	0.015	0.005	0.046	

Event rate and 95% CI

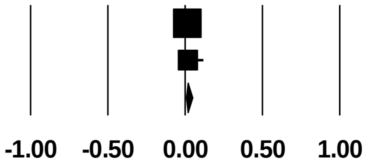

Dextrocardia among CHD

Study name | **Event rate and 95% CI**

Study name	Event rate	Lower limit	Upper limit	Total
Joshi A et al	0.014	0.003	0.054	2 / 144
Ramachandran U et al	0.018	0.003	0.118	1 / 55
Shah GS et al	0.036	0.012	0.105	3 / 84
	0.023	0.010	0.051	

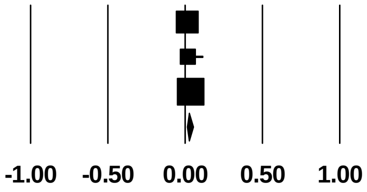

Total Anomalous Pulmonary Venous Connection among CHD

Study name | **Event rate and 95% CI**

Study name	Event rate	Lower limit	Upper limit	Total
Chapagain et al	0.012	0.002	0.079	1 / 85
Joshi A et al	0.014	0.003	0.054	2 / 144
Ramachandran U et al	0.018	0.003	0.118	1 / 55
Shah GS et al	0.036	0.012	0.105	3 / 84
Shah P et al	0.020	0.008	0.046	5 / 254
	0.020	0.012	0.036	

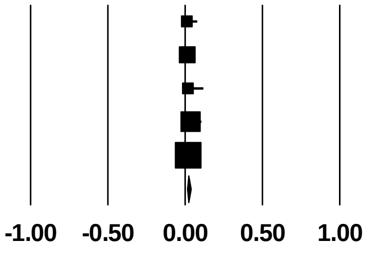

Double Outlet Right Ventricle among CHD

Study name	Event rate	Lower limit	Upper limit	Total
Chapagain et al	0.047	0.018	0.119	4 / 85
Joshi A et al	0.014	0.003	0.054	2 / 144
Shah P et al	0.039	0.021	0.072	10 / 254
	0.036	0.022	0.058	

Event rate and 95% CI

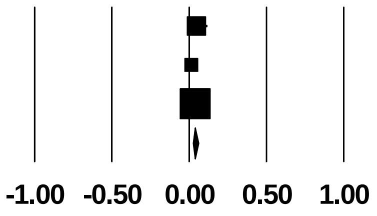

Transposition of the Great Arteries among CHD

Study name	Event rate	Lower limit	Upper limit	Total
Chapagain et al	0.024	0.006	0.089	2 / 85
Dongol SS et al	0.029	0.004	0.177	1 / 35
Joshi A et al	0.007	0.001	0.048	1 / 144
Ramachandran U et al	0.018	0.003	0.118	1 / 55
Shah GS et al	0.012	0.002	0.080	1 / 84
Shah P et al	0.031	0.016	0.062	8 / 254
	0.024	0.014	0.040	

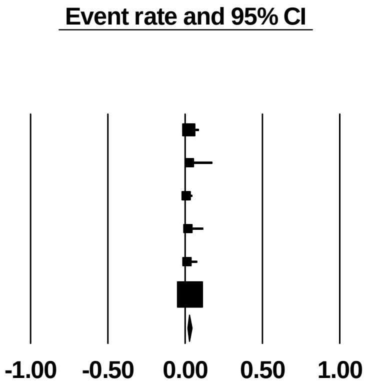

Truncus Arteriosus among CHD

Study name	Event rate	Lower limit	Upper limit	Total
Joshi A et al	0.014	0.003	0.054	2 / 144
Shah P et al	0.004	0.001	0.027	1 / 254
	0.009	0.003	0.028	

Event rate and 95% CI

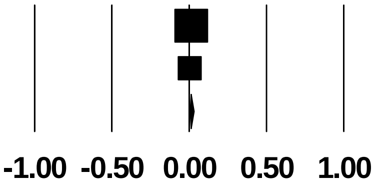

Complex Cardiac Defect among CHD

Study name	Event rate	Lower limit	Upper limit	Total
Chapagain et al	0.118	0.064	0.205	10 / 85
Dongol SS et al	0.314	0.183	0.483	11 / 35
Joshi A et al	0.063	0.033	0.116	9 / 144
Ramachandran U et al	0.073	0.028	0.178	4 / 55
Shrestha S et al	0.167	0.042	0.477	2 / 12
	0.125	0.062	0.236	

Event rate and 95% CI

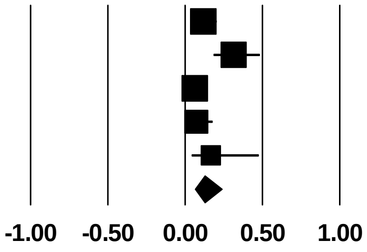

Difficult Breathing among CHD

Study name	Event rate	Lower limit	Upper limit	Total
Chapagain et al	0.518	0.412	0.622	44 / 85
Ramachandran U et al	0.164	0.087	0.286	9 / 55
Shah GS et al	0.690	0.584	0.780	58 / 84
	0.445	0.197	0.724	

Event rate and 95% CI

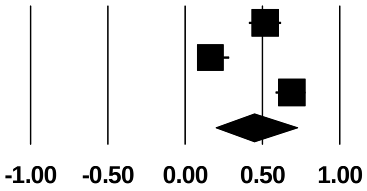

Cyanosis among CHD

Study name	Event rate	Lower limit	Upper limit	Total
Chapagain et al	0.118	0.064	0.205	10 / 85
Shah GS et al	0.202	0.130	0.302	17 / 84
	0.159	0.092	0.262	

Event rate and 95% CI

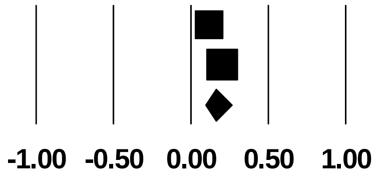

Failure to Thrive among CHD

Study name	Event rate	Lower limit	Upper limit	Total
Ramachandran U et al	0.236	0.142	0.366	13 / 55
Shah GS et al	0.119	0.065	0.207	10 / 84
	0.171	0.084	0.317	

Event rate and 95% CI

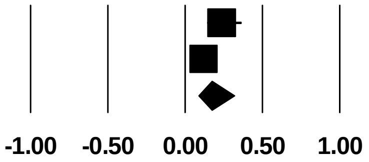

Developmental Delay among CHD

Study name	Event rate	Lower limit	Upper limit	Total
Ramachandran U et al	0.127	0.062	0.244	7 / 55
Shah GS et al	0.869	0.779	0.926	73 / 84
	0.498	0.023	0.977	

Event rate and 95% CI

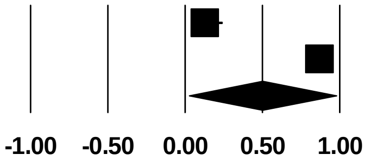

Respiratory Infection among CHD

Study name	Event rate	Lower limit	Upper limit	Total
Ramachandran U et al	0.582	0.449	0.704	32 / 55
Shah GS et al	0.524	0.418	0.628	44 / 84
	0.547	0.463	0.627	

Event rate and 95% CI

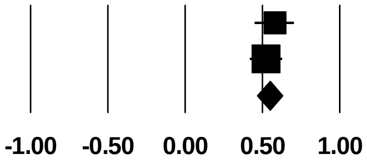

Supplement: Supplementary file 2 [file HSR2-6-e1147-s001.docx]
